# Complete genome sequence of the environmental *Burkholderia pseudomallei* strain 22-10884_313#20 from Guadeloupe, French West Indies

**DOI:** 10.1128/mra.00945-24

**Published:** 2025-03-28

**Authors:** Bernice J. M. Klotoe, Mégane Gasqué, Fabien Vorimore, Emma Newall-Rochelle, Vanina Guernier-Cambert, Karine Laroucau

**Affiliations:** 1 Animal Health Laboratory, Bacterial Zoonosis Laboratory, Paris Est University, ANSES 355169 , Maisons-Alfort, Île-de-France, France; 2 Institute of Ecology and Environmental Sciences-Paris (iEES-Paris), Sorbonne Université, UPEC, IRD, CNRS, INRAE 129930 , Paris, Île-de-France, France; 3 Identypath, Paris Est University, ANSES, Maisons-Alfort, France; 4 Faculty of Veterinary Technology, Kasetsart University 614849 , Bangkok, Bangkok, Thailand; University of Maryland School of Medicine, Baltimore, Maryland, USA

**Keywords:** *Burkholderia pseudomallei*, environmental, Guadeloupe

## Abstract

*Burkholderia pseudomallei* is the causative agent of melioidosis. Here, we present the complete genome sequence of the strain 22-10884_313#20*,* isolated from a soil sample in Guadeloupe (French West Indies).

## ANNOUNCEMENT

*Burkholderia pseudomallei* is a bacterium responsible for melioidosis, a serious tropical disease endemic to Southeast Asia and northern Australia. It thrives in contaminated soil or water and infects humans or animals by inoculation, inhalation, or ingestion (1). Clinical manifestations range from pneumonia to sepsis and skin infections, primarily affecting adults with comorbidities, particularly diabetes (2). Outside endemic areas, melioidosis is often underdiagnosed due to limited awareness and diagnostic challenges (3).

In the French West Indies, 25 cases of melioidosis have been reported since 1993 (4). Local contamination was suspected in patients without a travel history, but environmental *B. pseudomallei* was only recently confirmed with the isolation of strain 22-10884_313#20 from soil in Guadeloupe (4). Isolation involved liquid enrichment in erythritol and Ashdown media, plating on a modified chromogenic *Burkholderia cepacia* agar, and subculture on blood agar with 5% horse serum at 37°C for 4 days (4).

Genomic DNA from this strain was extracted for sequencing using two kits. For MinION sequencing, DNA was extracted using the Lucigen kit (Biosearch Technologies, UK), and libraries were prepared using the Rapid Barcoding SQK-RBK-004 kit (Oxford Nanopore Technologies, UK) without DNA shearing or size selection. Sequencing was performed on a MinION MK1B system using Flowcell R9.4.1, generating 25,025 reads with an N50 read length of 10,575 bp. Base calling was performed with Guppy (v6.5.7) using the model dna_r9.4.1_450bps_sup, and read analysis was performed with NanoPlot (v1.44.0) (5). Poor quality reads (10%) were filtered using Filtlong (v0.2.1) with parameters --keep_percent 90 and --mean_q_weight 10 (6). For MiSeq Illumina sequencing, DNA was extracted using the Roche kit (Roche Diagnostics, France). Libraries were prepared using the Illumina DNA Prep (M) Tagmentation kit (Illumina, USA) and sequenced on a MiSeq Illumina platform using the MiSeq Reagent kit v3 (2  ×  300). Sequencing generated 2,784,709 paired-end reads with 100× depth coverage. Quality assessment was performed using FastQC (v0.12.1) (7), and low-quality reads (<Q20) and adapters were removed using Fastp (v0.23.4) with the parameters --corrections and --cut_right (8).

Hybrid assembly using the Flye nano-raw model (v2.9.5) (9) produced two circular contigs with an N50 of 4,122,689 bp. Each contig formed a single unique graph edge, which was visualized using Bandage v0.9.0 (10) to confirm the circularization of the contigs. The draft assembly was corrected with long reads using the Medaka v1.11.3 (Oxford Nanopore Technologies Ltd). Illumina reads were aligned to the draft genome using BWA-MEM (v0.7.18) (11), with further polishing using Polypolish (12) and Pypolca (13). Genome quality was assessed using QUAST (v5.2.0) (14). Default parameters were used for the BWA-MEM aligner, Polypolish, Pypolca, and QUAST. Annotation was performed using NCBI’s PGAP (v2024-07-18.build7555) (15) with GeneMarkS-2+ (16).

The assembled genome of the strain is 7,364,047 bp in length, organized into two circular contigs (4,123,479 bp and 3,240,568 bp), corresponding to its two chromosomes. The annotation reveals 6,245 coding sequence genes, 235 pseudogenes, and 78 RNA genes. The genome has a G+C content of 67.94%, an N50 of 4,123,479 bp, and no undetermined bases (N).

Comparative genomic analysis of this strain with local and global strains may provide insights into the ecological and genomic adaptations of *B. pseudomallei* in the French West Indies.

## Data Availability

The genome sequence data of strain 22-10884_313#20 has been deposited at ENA under accession number GCA_964417475.1. MinION and Illumina reads have been deposited at ENA under accession numbers ERR13717737 and ERR13718582, respectively.
